# Case Report: From behavioral disorder to surgical emergency: a case of intussusception due to pica in an adolescent

**DOI:** 10.3389/fpsyt.2025.1690213

**Published:** 2025-11-07

**Authors:** Mohammed A. Alhaj, Mohamed Hassan Ahmed, Tala Jalkhi, Rama Armouche, Mira Amer Alrushdi, Hania Elmaghraby

**Affiliations:** 1 Department of Psychiatry, Sheikh Khalifa Medical City, Ajman, United Arab Emirates; 2 College of Medicine, Ajman University, Ajman, United Arab Emirates; 3 Department of Psychology, Zayed University, Dubai, United Arab Emirates; 4 Training & Development Department, Emirates Health Services, Sharjah, United Arab Emirates

**Keywords:** pica, intussusception, depression, CBT (cognitive behavioral therapy), surgery

## Abstract

**Background:**

Pica is a complex behavioral condition with both psychological and biological components, characterized by the persistent ingestion of non-nutritive substances. It often coexists with other mental health conditions, and when left untreated, may lead to life-threatening medical complications. While gastrointestinal obstruction is a known outcome, intussusception caused by pica is extremely rare, particularly in adolescents.

**Case presentation:**

We report a case of a 13-year-old Middle Eastern girl of mixed Emirati Indonesian descent, with a history of emotional distress and familial instability, who presented with persistent abdominal pain and vomiting. Imaging revealed an intussusception caused by foreign bodies extending from the stomach to the duodenum. The patient underwent emergency laparotomy, during which multiple ingested objects were removed, including hair ties, shoelaces, and a thin metal wire. A postoperative psychiatric evaluation led to a diagnosis of pica and major depressive disorder. Cognitive Behavioral Therapy (CBT) was initiated shortly after diagnosis, and the patient demonstrated significant clinical improvement, including cessation of pica behaviors without pharmacological intervention.

**Conclusion:**

This case demonstrates the rarity of intussusception as a surgical complication of pica and highlights the importance of early psychiatric assessment and coordinated multidisciplinary care. The patient’s recovery was facilitated by timely psychosocial intervention, with marked improvement following the introduction of CBT.

## Introduction

Pica is characterized by the chronic ingestion of non-food, non-nutritive substances and is often linked to underlying psychological distress or neurodevelopmental abnormalities. Although frequently classified as a psychiatric condition, the behavioral manifestations of pica can result in life-threatening medical complications if left untreated. The ingestion of foreign objects may lead to gastrointestinal toxicity, obstruction, or other serious outcomes. This case describes an uncommon and acute presentation of pica in a teenage girl with a history of emotional distress, dysfunctional family dynamics, and untreated major depressive disorder. Her persistent ingestion of hair ties and shoelaces culminated in an urgent surgical intervention for intussusception.

## Case presentation

A 13-year-old Middle Eastern girl was admitted on January 12, 2025, with a three-day history of abdominal pain and vomiting. On physical examination, her abdomen was soft, and her vital signs were within normal limits: temperature 36.7 °C, heart rate 85 bpm, respiratory rate 22 breaths per minute, blood pressure 113/70 mmHg, and oxygen saturation 98%. She was underweight, with a height of 156 cm, weight of 40.15 kg, and BMI of 16. An abdominal CT scan revealed intussusception caused by suspected foreign bodies extending from the stomach to the duodenum. Initial conservative management, including fluid resuscitation and close observation, was attempted in the emergency department; however, symptoms persisted, and surgical intervention was subsequently scheduled.

On January 13, 2025, the patient underwent a laparotomy involving multiple enterotomies and a gastrostomy, during which various foreign objects were removed, including hair ties, shoelaces, and a thin metal wire. The procedure was completed without complications.

Postoperatively, she remained clinically stable, tolerated oral intake, and showed no signs of infection or abdominal tenderness. Her recovery was uneventful, and she was monitored in the pediatric ward until January 20.

Due to the nature of the ingested materials and concerns about intentional behavior, a psychiatric consultation was initiated postoperatively. The patient disclosed that she had been living with her father and stepsiblings following the death of her mother in 2019. Both she and the household maid reported longstanding emotional distress, stemming from familial instability and psychosocial stressors. The patient described persistent low mood, frequent crying spells, and social withdrawal. She had begun ingesting hair ties approximately three years earlier as a means of coping with stress. Eight months prior to admission, she developed suicidal ideation without taking action and engaged in self-harming behaviors using sharp objects, resulting in visible scars. She denied any suicidal intent or substance use.

During psychiatric evaluation, she was cooperative and maintained good eye contact, with normal speech and behavior. Her affect was low, but there were no signs of hallucinations or delusions. Cognition, insight, and judgment were intact. Standardized assessments were administered, yielding the following scores: Beck Depression Inventory (BDI) – 32 (severe depression), Beck Anxiety Inventory (BAI) – 44 (severe anxiety), and Yale-Brown Obsessive Compulsive Scale (Y-BOCS) – 22 (moderate OCD symptoms). Risk assessment revealed no immediate threat to self or others. Based on clinical evaluation and standardized tools, she was diagnosed with pica and major depressive disorder, as per DSM-5 criteria. Psychoeducation and reassurance were provided, and a follow-up plan for outpatient psychiatric care was arranged.

She was discharged on January 20, 2025, with continued management of postoperative pain and constipation.

The patient’s physical recovery remained stable. An abdominal X-ray performed on February 24, 2025, revealed a normal gas pattern, without air-fluid levels, free air, or radio-opaque shadows, indicating no residual surgical complications. However, she continued to report constipation, prompting referral to pediatric gastroenterology. Additional referrals were made to gynecology for excessive menstrual bleeding and to dermatology for acne vulgaris. Her nutritional workup showed ferritin at 19.1 pmol/L, at the lower limit of normal, and zinc at 82.06 µg/dL (reference range: ≥44 µg/dL), indicating adequate micronutrient levels. Dermatological evaluation attributed her acne to inadequate skin hygiene rather than nutritional or endocrine causes, while gynecological assessment found no underlying abnormalities. Constipation improved with dietary regulation and behavioral modification.

**Figure 1:**
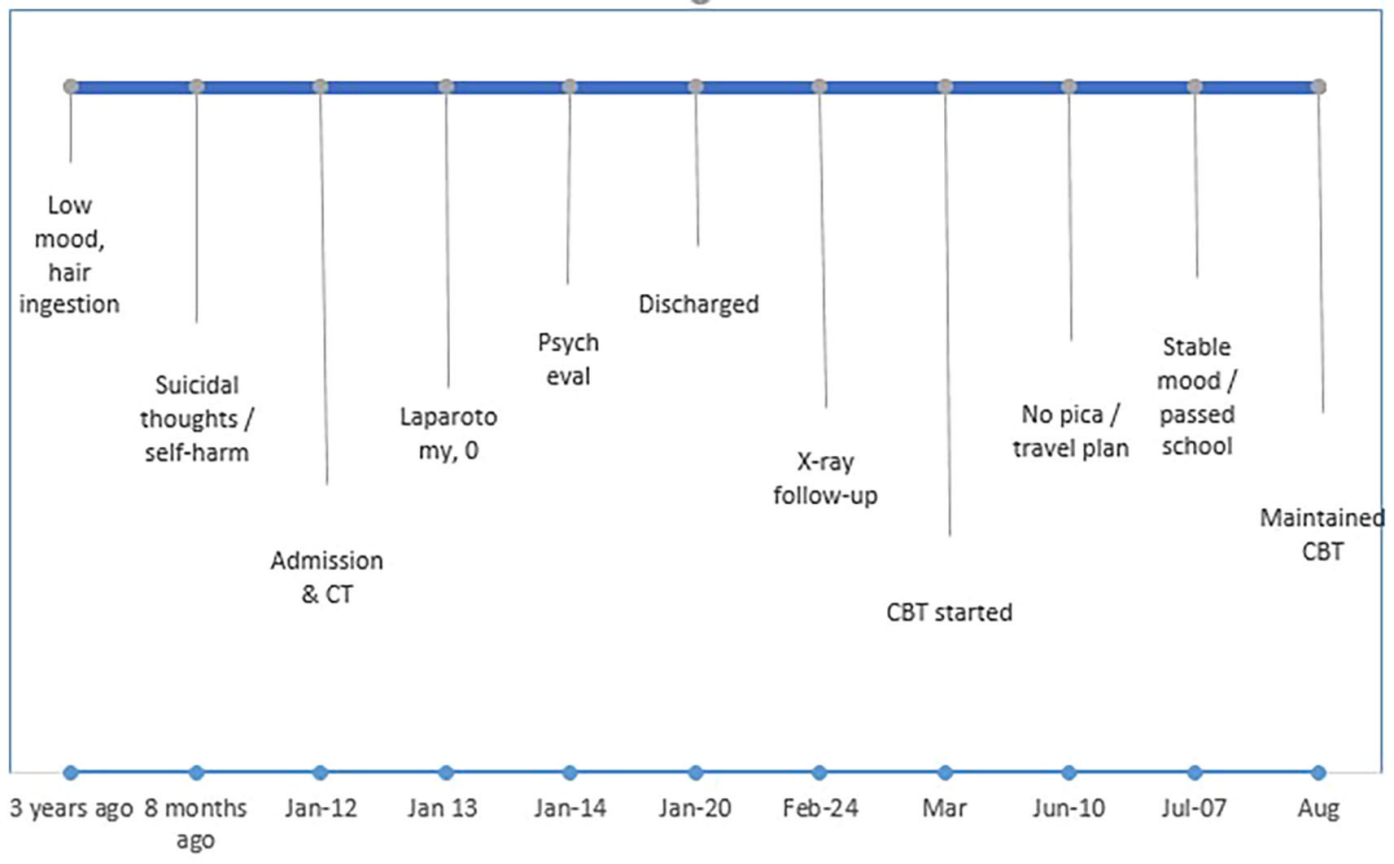
Clinical timeline of the case.

**Figure 2:**
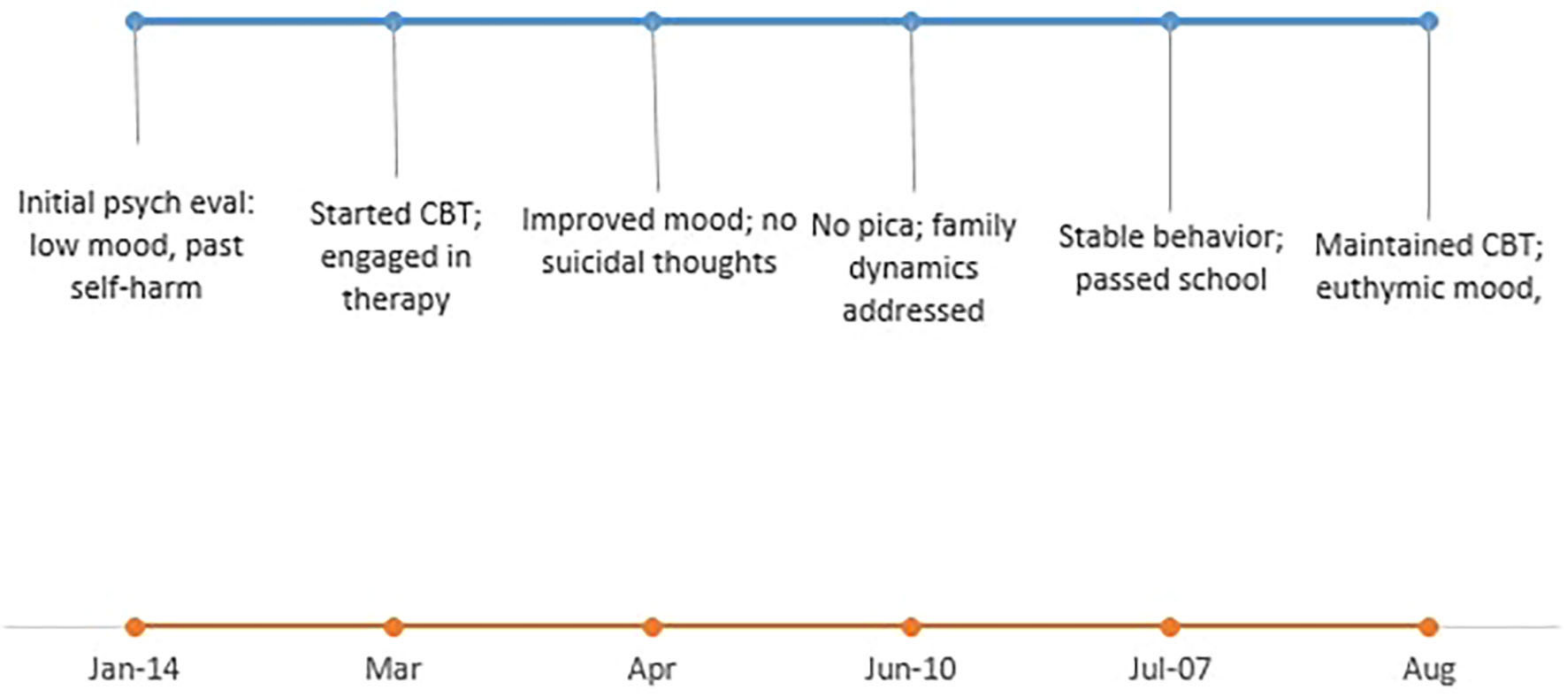
Psychiatric and behavioral progress over time.

During psychiatric follow-up, Cognitive Behavioral Therapy (CBT) was initiated as outpatient care, without pharmacological treatment. The patient showed steady clinical improvement, including mood stabilization and cessation of pica behavior (**Figures 1**, **2**). She denied ongoing suicidal ideation or self-harm. In one follow-up visit, her father attended and shared additional context regarding family dynamics, including emotional unavailability, unstable personal relationships, and alcohol use—factors contributing to her long-term emotional distress.

The patient’s surgical wound healed well, and subsequent abdominal examinations were unremarkable. A multidisciplinary care approach—encompassing surgery, psychiatry, pediatrics, gastroenterology, gynecology, and dermatology—proved essential in addressing both the acute and underlying conditions. Her long-term management plan emphasized continued psychiatric follow-up, structured psychotherapy, and strengthened family support.

Goals included preventing relapse, enhancing emotional regulation, and addressing the psychosocial contributors to her behavioral dysregulation.

## Intervention and psychotherapeutic management

Following the diagnosis of pica and major depressive disorder, the patient was enrolled in outpatient psychotherapy without pharmacological treatment. The therapeutic approach was grounded in Cognitive Behavioral Therapy (CBT) and focused on addressing both the behavioral symptoms and underlying emotional distress.

CBT was initiated in March 2025, and the patient completed four sessions of cognitive restructuring prior to relocation in June 2025 (**Figure 2**). Each session lasted approximately 45–50 minutes and targeted persistent negative automatic thoughts related to low self-esteem, self-blame, and hopelessness. Through guided exercises and therapist-facilitated reflection, she learned to identify maladaptive beliefs and replace them with more adaptive, reality-based alternatives. These sessions aimed to enhance self-awareness, build self-efficacy, and improve emotional regulation.

In parallel, psychoeducation was provided to the patient’s father to increase his understanding of the emotional and behavioral impact of neglectful caregiving, unresolved grief, and ongoing family instability. This intervention addressed the role of parental availability and support in adolescent mental health, while also promoting insight into the consequences of his own inconsistent involvement and alcohol misuse.

As part of the family-focused intervention, the patient was relocated in June 2025 to live with her paternal aunt, a retired teacher who was aware of her brother’s behavioral challenges. This move provided a more stable and supportive environment, marking a pivotal point in her recovery. The aunt implemented several positive adjustments in the patient’s life, including improved self-hygiene routines, structured academic support, and social enrichment activities such as family outings and travel. In-person follow-up continued until August 2025, during which the patient maintained behavioral stability, emotional improvement, and full remission of pica symptoms.

Following these interventions, the pica behaviors ceased entirely, with the exception of a single episode during a family gathering where sensitive family issues were discussed. The brief relapse episode, in which the patient chewed a hairband, appeared to be triggered by emotional distress during the gathering that reminded her of prior stressful experiences within her household. This situational cue likely reactivated conditioned associations between anxiety and oral self-soothing behaviors, consistent with stress-induced regression mechanisms described in pica and related impulse-control disorders. On that occasion, the patient chewed, but did not swallow the hairband, reflecting a markedly diminished behavioral response to psychological stress. Understanding such context-dependent triggers is essential in relapse prevention, as it highlights the role of emotional memory and environmental cues in symptom re-emergence.

Overall, the therapeutic response was favorable, and no pharmacologic treatment was required.

The combination of trauma-informed CBT, family restructuring, and psychoeducation yielded clinically meaningful improvement in mood, coping strategies, and behavioral regulation.

## Discussion

This case highlights a rare but serious presentation of pica, where persistent ingestion of non-food items—hair ties and shoelaces—served as a maladaptive coping mechanism amid familial stress and emotional distress, ultimately causing intussusception requiring surgical intervention. Psychiatric care was essential to address the underlying behavioral and mood disorders.

According to DSM-5, pica involves persistent ingestion of non-nutritive substances for at least one month, developmentally inappropriate and not culturally normative. It commonly involves items like dirt, hair, or ice, and may be linked to nutritional deficiencies such as iron anemia.

Pica can co-occur with neurodevelopmental disorders or psychiatric conditions and warrants independent clinical attention (1–3).

Neurobiologically, pica shares features with compulsive disorders and impulse control deficits. Dysfunction in cortico-striatal-thalamo-cortical circuits and dysregulated dopamine signaling in prefrontal regions impair behavioral inhibition and gratification delay, aligning pica with conditions such as ADHD and binge-eating disorder (4–6). The behavior may initially provide sensory pleasure and emotional relief, reinforcing compulsive ingestion via dopamine-mediated reward pathways, eventually shifting into habitual automaticity akin to substance use disorders (6, 7).

Neuroimaging implicates altered activity in brain areas responsible for reward processing, impulse control, emotional regulation, and interoception—including the ventral tegmental area, nucleus accumbens, prefrontal cortex, insula, anterior cingulate cortex, and amygdala—which collectively contribute to the persistence of pica behaviors and stress vulnerability (6, 7).

Psychologically, pica often functions as a maladaptive coping mechanism for emotional distress, anxiety, or boredom, sharing characteristics with self-injurious behaviors like NSSI by providing sensory grounding or emotional numbing without explicit intent to self-harm (8, 9). In this case, the patient used ingestion to manage overwhelming family stress rather than deliberate self-injury.

Childhood trauma, adverse experiences, and family dysfunction significantly increase vulnerability to pica by disrupting emotional regulation and fostering compulsive coping behaviors. Insecure attachment patterns further exacerbate these risks, with trauma-informed frameworks viewing pica as an adaptive response to dysregulated arousal and emotional pain (10–12).

The patient’s mixed Emirati–Indonesian background may have shaped both symptom expression and family attitudes toward psychiatric care. In Middle Eastern societies, mental health concerns are often associated with stigma, leading families to initially attribute behavioral changes to stress or spiritual causes rather than psychological disorders. Similarly, collectivist cultural values emphasizing family privacy may delay professional help-seeking until symptoms become severe. Awareness of these cultural dynamics is crucial for clinicians working in the Gulf region, as culturally sensitive psychoeducation and family engagement can significantly enhance adherence and reduce relapse risk.

Though gastrointestinal complications from pica are uncommon, they can be life-threatening, as shown in reported cases involving bezoars, obstruction, and intussusception requiring surgical intervention, especially among individuals with neurodevelopmental or psychiatric comorbidities (13–15). Our patient’s successful multidisciplinary care adds to this body of evidence.

While this comparative analysis provides valuable insights into the psychiatric and medical management of pica, certain limitations should be acknowledged. As this report only details one case, its findings cannot be generalized to all patients with pica. Furthermore, relying on self-reported ingestion history may result in recall bias or underreporting, especially given the behavioral and emotional elements at play Nonetheless, the consistency of clinical findings, imaging results, and surgical outcomes supports the validity of the observed associations.

Comparative analysis of similar cases reveals variability in ingestion patterns, psychiatric comorbidities, and treatment responses. Notably, our adolescent patient achieved remission of pica behaviors and mood stabilization with cognitive behavioral therapy (CBT) alone, within a multidisciplinary framework—a contrast to cases requiring pharmacotherapy or limited psychiatric follow-up.

Routine psychiatric screening and integrated multidisciplinary management—including psychiatry, pediatrics, surgery, and nutrition—are critical to improve diagnosis, prevent complications, and optimize outcomes (16–19). Evidence-based psychotherapeutic approaches (CBT, trauma-focused therapies) combined with pharmacological treatments for comorbid mood and anxiety disorders and nutritional supplementation (especially iron) constitute the foundation of effective care.

Despite growing awareness of pica’s medical risks, it remains under recognized as an early behavioral indicator of serious psychological morbidity. This case underscores the importance of early identification and intervention in vulnerable youth to prevent progression to severe medical and psychiatric complications and to improve long-term prognosis.

## Conclusion

Pica, though primarily considered a behavioral and psychiatric condition, can result in life-threatening medical complications when left unrecognized. >This case demonstrates a rare and severe surgical consequence of pica, manifesting as intussusception caused by the ingestion of hair ties and shoelaces. Emergency laparotomy was required, followed by comprehensive psychiatric evaluation, which revealed a comorbid major depressive disorder.

Early initiation of cognitive behavioral therapy (CBT) resulted in rapid and sustained improvement, with cessation of pica behaviors and mood stabilization, without the need for pharmacologic treatment. This outcome underscores the critical importance of timely psychiatric assessment in adolescents presenting with unusual behavioral symptoms, particularly those with histories of trauma, loss, or neglect (20, 21).

The case also highlights the effectiveness of a multidisciplinary approach—including psychiatry, surgery, pediatrics, gastroenterology, dermatology, and family intervention—in promoting recovery and preventing relapse. Additionally, it brings attention to an underexplored area in the literature: the potential of pica as an early behavioral indicator of significant psychological or emotional distress. Although this case suggests that pica behaviors may reflect early signs of psychosocial disturbance, establishing this connection requires longitudinal and multi-center studies. Further research is needed to evaluate whether early identification of pica could improve risk stratification and guide preventative mental health interventions in vulnerable populations (22, 23).

## Data Availability

The original contributions presented in the study are included in the article/**Supplementary Material**. Further inquiries can be directed to the corresponding author.

## References

[1] American Psychiatric Association . Diagnostic and statistical manual of mental disorders. 5th ed. Washington, DC: American Psychiatric Association (2022).

[2] Al Nasser Y Muco E Alsaad AJ . Pica. In: StatPearls. StatPearls Publishing, Treasure Island (FL (2023). Available online at: https://www.ncbi.nlm.nih.gov/books/NBK537324/., PMID: 30335275

[3] Lagusis B Solomon SS Ruther S Olson LB . When pica takes a step too far: Small bowel obstruction due to ingested shoe insoles, a case report and review of literature. J Surg Case Rep. (2025) 2025:rjaf243. doi: 10.1093/jscr/rjaf243, PMID: 40297717 PMC12036824

[4] Everitt BJ Robbins TW . Drug addiction: Updating actions to habits to compulsions ten years on. Annu Rev Psychol. (2015) 67:23–50. doi: 10.1146/annurev-psych-122414-033457, PMID: 26253543

[5] Fields VL Soke GN Reynolds A Tian LH Wiggins L Maenner M . Pica, autism, and other disabilities. Pediatrics. (2021) 147:e2020007245. doi: 10.1542/peds.2020-0462, PMID: 33408069 PMC9188765

[6] Gundogar D Demir SB Eren I . Is pica in the spectrum of obsessive-compulsive disorders? Gen Hosp Psychiatry. (2003) 25:293–4. doi: 10.1016/s0163-8343(03)00039-2, PMID: 12850663

[7] Huremović D Nagalla ML Khan S . Allotrichophagia: A unique case of parental adjustment to filial pediatric Malignancy. Case Rep Psychiatry. (2022) 2022:5949321. doi: 10.1155/2022/5949321, PMID: 35755004 PMC9217604

[8] Fineberg NA Potenza M Chamberlain SR Berlin HA Menzies L Bechara A . Probing compulsive and impulsive behaviors, from animal models to endophenotypes: A narrative review. Neuropsychopharmacology. (2010) 35:591–604. doi: 10.1038/npp.2009.185, PMID: 19940844 PMC3055606

[9] Kim A Patel A Loomis JG Okwara T Miller M . An unusual case of trichotillomania and trichophagia associated with authentic hair extension as seen in a young African-American female adult. Discov Psychol. (2022) 2:46. doi: 10.1007/s44202-022-00053-3

[10] Burke M . Pica: Practice essentials, background, etiology (2024). Medscape. Available online at: https://emedicine.medscape.com/article/914765-overviewa1.

[11] Moline R Hou S Chevrier J Thomassin K . A systematic review of the effectiveness of behavioural treatments for pica in youths. Clin Psychol Psychother. (2020) 28:39–55. doi: 10.1002/cpp.2491, PMID: 32628326

[12] Attia M Lavoie-Forrest AA Langius P Melnitsky L Lopez S . Gastroduodenal obstruction secondary to pica-associated bezoar: A case report. Clin Pract cases Emerg Med. (2025) 9:53–6. doi: 10.5811/CPCEM.21300, PMID: 39903611 PMC12509944

[13] Schnitzler E . The neurology and psychopathology of pica. Curr Neurol Neurosci Rep. (2022) 22:531–6. doi: 10.1007/s11910-022-01218-2, PMID: 35674869

[14] Svaldi J Griepenstroh J Tuschen-Caffier B Ehring T . Emotion regulation deficits in eating disorders: A marker of eating pathology or general psychopathology? Psychiatry Res. (2012) 197:103–11. doi: 10.1016/j.psychres.2011.11.009, PMID: 22401969

[15] Teicher MH Samson JA . Annual research review: Enduring neurobiological effects of childhood abuse and neglect. J Child Psychol Psychiatry. (2016) 57:241–66. doi: 10.1111/jcpp.12507, PMID: 26831814 PMC4760853

[16] Najmi S Wegner DM Nock MK . Thought suppression and self-injurious thoughts and behaviors. Behav Res Ther. (2006) 45:1957–65. doi: 10.1016/j.brat.2006.09.014, PMID: 17074302 PMC2211522

[17] O’Malley PG Wong PWK Kroenke K Roy MJ Wong RKH . The value of screening for psychiatric disorders prior to upper endoscopy. J Psychosom Res. (1998) 44:279–87. doi: 10.1016/S0022-3999(97)00250-X, PMID: 9532557

[18] Putnam KT Harris WW Putnam FW . Synergistic childhood adversities and complex adult psychopathology. J Trauma Stress. (2013) 26:435–42. doi: 10.1002/jts.21833, PMID: 23893545

[19] Ross WJ Chan E Rappaport LA . Pediatrician-psychiatrist collaboration to care for children with ADHD, depression, and anxiety. Clin Pediatr. (2011) 50:37–43. doi: 10.1177/0009922810379499, PMID: 20724316

[20] Bhatia MS Gupta R . Pica responding to SSRI: An OCD spectrum disorder? World J Biol Psychiatry. (2009) 10:936–8. doi: 10.1080/15622970701308389, PMID: 17853279

[21] Brewerton TD . Eating disorders, trauma, and comorbidity: Focus on PTSD. Eat Disord. (2007) 15:285–304. doi: 10.1080/10640260701454311, PMID: 17710567

[22] Guinan D Drvar T Brubaker D Ang-Rabanes M Kupec J Marshalek P . Intentional foreign body ingestion: A complex case of pica. Case Rep Gastrointest Med. (2019) 2019:7026815. doi: 10.1155/2019/7026815, PMID: 30881707 PMC6381557

[23] Liu B Jiang L Yuan M Zhu H Zhang W . Pica in a girl with non-suicidal self-injury: A case report. Front Psychiatry. (2023) 14:1320079. doi: 10.3389/fpsyt.2023.1320079, PMID: 38179246 PMC10765586

