# Supplementary figures and images for: Case Report: From behavioral disorder to surgical emergency: a case of intussusception due to pica in an adolescent
Source: Front Psychiatry. 2025 Nov 7;16:1690213. doi: 10.3389/fpsyt.2025.1690213 (PMC12634625; doi:10.3389/fpsyt.2025.1690213)

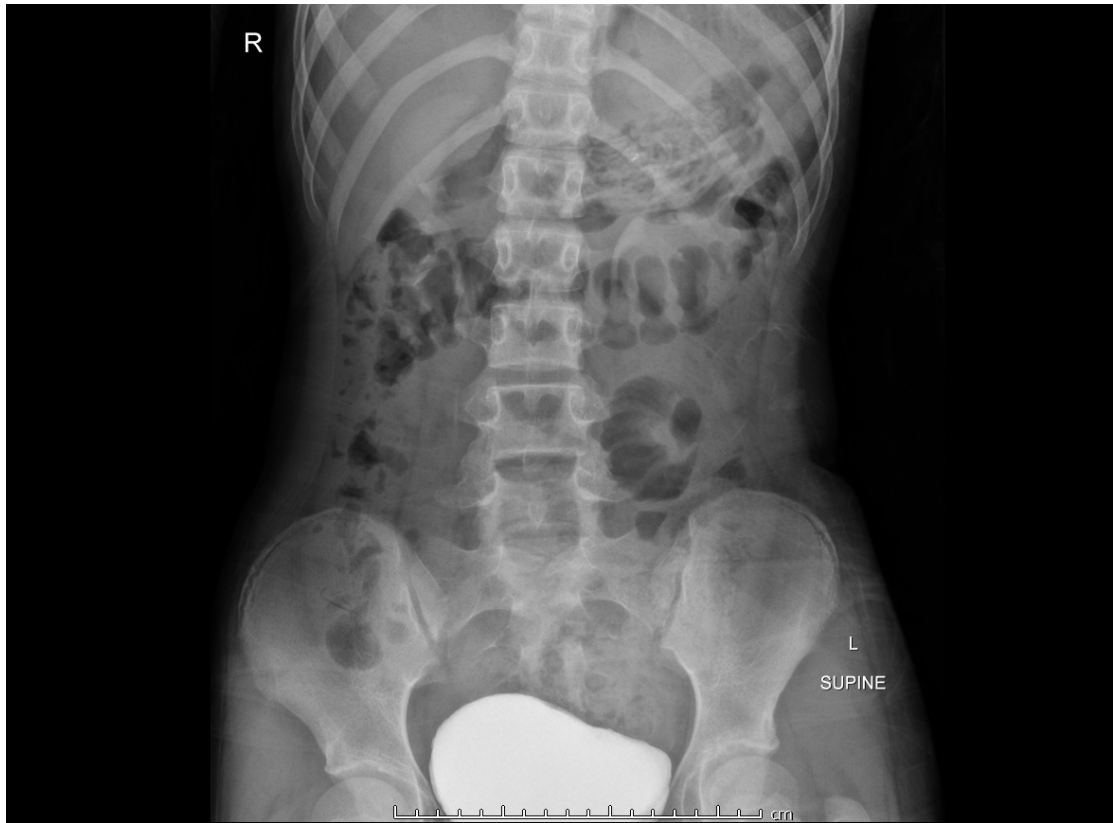

Supplement: Supplementary file 1 [file Image1.pdf]
